# Information exchange or discussion? A qualitative study on cross-sectoral collaboration between social security service and healthcare service for patients with chronic fatigue

**DOI:** 10.1186/s12913-025-13857-5

**Published:** 2025-12-09

**Authors:** Maryam Haghshenas, Aslak Steinsbekk, Tormod Landmark, Astrid Woodhouse, Karen Walseth Hara

**Affiliations:** 1 Department of Public Health and Nursing, Faculty of Medicine and Health Sciences, Norwegian University of Science and Technology (NTNU), Trondheim, 7491 Norway; 2 Norwegian Centre for E-Health Research, Tromsø, Norway; 3 Department of Circulation and Imaging, Faculty of Medicine and Health Sciences, Norwegian University of Science and Technology (NTNU), Trondheim, Norway; 4 Department for Pain and Complex Disorders, St. Olavs Hospital, Trondheim University Hospital, Trondheim, Norway; 5 The Norwegian Labour and Welfare Administration of Trøndelag (NAV), Trondheim, Norway

**Keywords:** Chronic fatigue, Cross-sectoral collaboration, Qualitative, Social security, Specialist healthcare

## Abstract

**Background:**

Chronic fatigue, with or without an identified underlying cause, can severely restrict social participation, education, employment, and daily activities, often leading to long-term disability. In parallel with the health challenges, many affected individuals also seek support from social security services. While cross-sectoral collaboration between healthcare and social security service in managing chronic conditions is increasingly studied, little is known about such collaboration for patients with chronic fatigue. The aim of this study was therefore to investigate the experiences of patients with chronic fatigue of unclarified cause and social security supervisors regarding cross-sectoral collaboration between healthcare and social security services in Norway, particularly through collaborative meetings initiated by specialist healthcare service.

**Methods:**

A qualitative study was conducted using semi-structured individual interviews with nine patients and five social security supervisors who had participated in at least one collaborative meeting initiated by a tertiary care pain clinic at which the patients were referred to for a multidisciplinary examination. The meetings were part of a proposed care pathway for patients with chronic fatigue of unclarified cause and involved general practitioners, specialist healthcare providers, and social security supervisors. The data was analyzed using thematic analysis.

**Results:**

It was found that the informants experienced pre-existing relationships with social security supervisors influenced the quality of collaboration. Some had not had any contact before the first collaborative meeting. The collaborative meetings varied in depth, from basic information sharing to meaningful dialogue and planning. The meeting structure, timing of participation, and digital format affected engagement and flow. The follow-up processes were often unclear, with varying expectations about responsibilities. While informants generally valued the meetings, they emphasized that effective collaboration required continuity, preparation, and sustained engagement beyond the meeting.

**Conclusion:**

Collaborative meetings between healthcare and social security service were perceived as beneficial for managing complex cases of chronic fatigue. However, their effectiveness is perceived to depend on relational continuity, inclusive dialogue, and clear follow-up procedures. Structural adjustments, such as improved preparation, consistent supervisory relationships, and facilitation of shared decision-making, are suggested as actions to enhance the collaborative process and ensure person-centered cross-sectoral support for this patient group.

**Supplementary Information:**

The online version contains supplementary material available at 10.1186/s12913-025-13857-5.

## Background

Chronic fatigue is associated with a variety of diseases and is common in the general population also in the absence of identified underlying pathology [1]. Myalgic Encephalomyelitis/Chronic Fatigue Syndrome (ME/CFS) is a diagnosis commonly applied to cases of medically unexplained, disabling fatigue that is typically accompanied by additional symptoms such as cognitive impairments and sleep disturbances [2, 3]. In this and other conditions characterized by chronic fatigue, social participation, educational engagement, employment, and everyday activities are severely restricted, often leading to long-term disability [1, 4–8]. A predominance of women with chronic fatigue in the general population has been consistently reported [9, 10]. Despite its impact, people with chronic fatigue syndrome frequently experience marginalization in both healthcare and welfare systems, often encountering disbelief, inadequate support, and a lack of recognition of their condition [11].

Within healthcare, conditions characterized by chronic fatigue remain underprioritized, in part due to the absence of definitive biomarkers and historical controversy surrounding their classification [3, 12, 13]. Physicians report uncertainty about how to diagnose or manage patients’ symptoms, which contributes to delayed diagnoses and limited access to effective care [2, 14]. Clinical encounters in this patient group are often reported to be poor, and marked by skepticism or dismissal, leading to frustration and loss of trust in health professionals [11, 15–18].

In parallel with their health challenges, patients often seek support from social security services, including access to sickness or work disability benefits, work assessments, or social support programs [19, 20]. Yet, welfare systems frequently rely on structured assessment protocols and functional evaluations that have been reported to be ill-suited to non-visible conditions like those dominated by chronic fatigue [11]. Patients can experience denials, inconsistent decision-making, and high administrative burdens, all of which can worsen symptoms and psychological distress [21].

To address these complex and overlapping health and social security needs, improved coordination and collaboration between healthcare service and social security service is increasingly promoted as a strategy for enhancing support for individuals with long-term health conditions [22, 23]. Examples of such collaboration may include joint assessments, shared care plans, interdisciplinary meetings, or information exchange mechanisms. These efforts aim to create coordinated, person-centered services that reduce gaps between sectors and increase the likelihood of sustainable support [24]. Collaborative meetings, in particular, have been identified as a valuable arena for better information sharing, creating shared understanding and opportunities for building trust between sectors [25, 26]. However, effective collaboration is difficult to achieve in practice. Healthcare and welfare institutions often operate with different institutional logics, where healthcare tends to prioritize symptoms and diagnosis, while social security frameworks focus on function, prognosis, and eligibility. As a result, collaboration may be challenged by these differing dynamics [27].

While there is growing literature on cross-sectoral collaboration in managing chronic conditions, particularly mental health and musculoskeletal disorders [28, 29], research on collaboration in the context of patients with chronic fatigue is limited.

The aim was therefore to investigate the experiences of patients with chronic fatigue of unclarified cause and social security supervisors regarding cross-sectoral collaboration between healthcare and social security services in Norway, particularly through collaborative meetings initiated by specialist healthcare service.

## Methods

A qualitative study using semi-structured individual interviews was conducted between September 2024 and February 2025. The Consolidated Criteria for Reporting Qualitative Research (COREQ) was consulted in reporting of the study [30]. The study was approved by the Norwegian Agency for Shared Services in Education and Research, which ensures legal access to necessary personal data for research (489407). The study was conducted by a team of five researchers (three female and two male).

### Setting

This study was conducted within a research project at the tertiary care outpatient multidisciplinary pain center at St. Olavs Hospital in Trondheim in Central Norway. The pain center offers multidisciplinary assessment and sometimes follow-up for patients with chronic pain or chronic fatigue, in which measures from primary care, mostly by general practitioners, or organ-specific specialists have not been adequate. The research project aims to improve coordination and support for patients with chronic fatigue, who are referred to the pain center by their general practitioners for evaluation of diagnosis, rehabilitation potential, and prognosis, by developing and testing a new care pathway, only introduced in the pain center in central Norway [31]. The most prominent change to previous organization of care was the introduction of video-based meetings involving the patients, their next-of-kind if desired, specialist healthcare providers, and general practitioners. In addition, social security service supervisors were also invited to the video-based meeting if patients had or were expected to require work-related support or social benefits. The meetings aimed to address this need by bringing together relevant actors to jointly evaluate patient situations and coordinate follow-up measures.

While the meetings were initially intended to be digital, planning also took into account patients’ preferences to participate either digitally or in person from the pain center or their general practitioners’ office. When patients opted for digital participation, the meetings were conducted entirely digitally. In other cases, healthcare providers working at the same location, i.e. the pain center, could join from the same screen.

Participation of social security supervisors required patient consent and was typically arranged so that supervisors joined the meeting after the clinical summary had been presented, usually midway through the meeting. The research project was responsible for piloting the care pathway at the pain clinic in Central Norway, where the focus of this study was only on the meetings where the social security supervisors were involved, making it a cross-sectoral collaboration between social security and healthcare service.

In Norway, these sectors operate under distinct organizational structures, mandates, and legal frameworks. The Norwegian Labour and Welfare Administration (NAV), hereby referred to as social security service, a joint state and municipal agency, is responsible for administering social benefits and facilitating individuals’ participation in the workforce [32]. Healthcare services, publicly funded and universally accessible, are delivered at both municipal and specialist levels [33]. Social security supervisors working at NAV have almost exclusively collaborated with the healthcare sector through general practitioners, who provide medical documentation and health-related information for the supervisors’ case processing [34]. However, there has been a recent shift towards closer and more integrated collaboration for complex cases such as patients with chronic fatigue [31].

### Participants and recruitment

The inclusion criteria were patients enrolled in the chronic fatigue care pathway and social security supervisors who participated in at least one cross-sectoral collaborative meeting initiated by the pain clinic. Patients and supervisors were not required to participate in the same collaborative meeting. The informants were recruited using purposive sampling. To ensure maximum variation in informants’ characteristics, recruitment targeted individuals from various age groups and work situations for patients, and variety in age, gender and work experience for supervisors, with the aim of reflecting a broad range of experiences within each group.

Recruitment was facilitated through the clinic’s administrative staff, who informed eligible participants about the study prior to each meeting. This was done by sending out practical information, including how the study would be conducted, what participation would involve, and contact information for the first author and the clinic coordinator. In addition, patients and supervisors were reminded about the study at the end of each meeting and were asked whether the coordinator\ the first author could contact them. Individuals who were interested or wanted more information were then either asked to contact the clinic or the first author themselves or consented to being contacted by them. Those who expressed interest were subsequently sent an information letter and a formal invitation to participate, distributed by the first author. Informed consent was obtained from all participants prior to the interview.

### Data collection

Data was collected using semi-structured individual interviews conducted by the first author (female), who was a PhD candidate at the time of the study with experiences in conducting qualitative studies. The researcher did not have any prior relationship with any of the informants. The interviews took place either physically at the pain clinic, or digitally using Teams, depending on the participants’ geographical location, medical condition, or preference. The interviews lasted between 30 and 60 min with a median of 47 min, and were audio recorded. The recordings were transcribed by an authorized transcription company. To ensure the accuracy of the transcripts, the first author reviewed all transcripts by reading them while listening to the recording and correcting any errors. Data collection and analysis were carried out in parallel. Interviewing ceased when the authors determined that the collected data provided sufficient diversity of experiences to address the research question and the final interviews with both patients and supervisors reflected similar topics to those raised by earlier informants, without the emergence of new themes [35].

The interviews were conducted first with the patients and then with the supervisors, in order to explore the supervisors’ perspectives on the patients’ experiences. This sequencing allowed for deeper exploration of patient-identified issues and provided insights into how supervisors interpreted and addressed similar challenges in practice. For the patients, a semi-structured guide was developed for this study based on literature and discussions among the authors. The main question was the informants’ experiences of the collaboration between social security service and healthcare service in general and in the collaborative video meetings in particular. If not spontaneously mentioned, informants were also asked about work participation, digital collaboration, challenges encountered, and suggestions for improvement (See supplementary file 1).

Following analysis of the patient interviews by three of the authors (MH, AS, and KWH), the findings were used to develop the interview guide for social security supervisors. In addition to being asked about their experiences of collaborative meetings at the pain clinic, the supervisors were invited to reflect on cross-sectoral collaboration with specialist healthcare more broadly, and to comment on patient experiences shared during the meetings. The patients’ experiences mentioned were, among other things, having to change social security supervisors several times, experiencing the meetings as primarily for information exchange rather than treatment planning, and lacking clear follow-up from social security afterwards. Some experienced feeling uncomfortable sharing personal feelings in this setting, reported feeling outnumbered or overpowered, being one patient facing several professionals (See supplementary file 2).

### Data analysis

The data were analyzed following the Systematic Text Condensation method, consisting of the following four steps: (1) total impression (2), identifying and sorting meaning units (3), condensation, and (4) synthesizing [36]. This process was done iteratively and parallel to the data collection. The software program NVivo was used during the analysis process.

The analysis process began with the first author selecting four interviews (2 patients and 2 supervisors) from those conducted at the time for all authors to read and reflect upon. Four preliminary themes were agreed upon among the authors at this stage (Different relationships affecting the meeting, Framework for meetings, information exchange or discussion point?, and Follow-up measures). Following this, the first author continued the analysis by reading through the remaining interviews, identifying meaning units, and organizing them under the agreed-upon themes. Although the data from supervisors’ interviews were mostly reflections on how the patients experienced the meetings, in some cases the supervisors’ experiences were presented independently when these were not covered by the patients. These meaning units were then condensed, with careful attention to maintaining the informants’ original expressions to ensure an accurate representation of their perspectives.

Throughout the process, and as the rest of the interviews were conducted, the authors engaged in multiple rounds of discussion. These discussions continued until a consensus on the final four themes presented in the results section was reached. In the final step, the condensed material was synthesized into generalized descriptions that formed the narrative of the results. Illustrative quotations were selected by the first author, who translated them into English and the translations were reviewed and revised by the co-authors. To further ensure accuracy, a native English speaker fluent in Norwegian also reviewed the translations.

## Results

In total, 14 informants were included, of whom 9 were patients, and 13 were female (Tables 1 and 2).

**Table 1:** Characteristics of the patients (P)(*N* = 9)

Characteristics	*N*
**Gender**	
Female	9
**Age**	
< 30	4
30–39	2
40–49	2
> 50	1
**Work situation**	
Not working	6
Part-time working	3
**Contact with supervisor before the meeting**	
Had prior contact	6
No prior contact	3

**Table 2:** Characteristics of the social security supervisors (S)(*N* = 5)

Characteristics	*N*
**Gender**	
Male	1
Female	4
**Age**	
< 30	1
30–39	3
40–49	1

The findings were categorized into four themes: “Relation to social security supervisor affecting the meeting”, “Information exchange or discussion?”, Dynamics of collaborative meetings”, and “Collaboration beyond the meeting”.

Across these themes, the informants talked about the complexity of collaboration between social security service and healthcare service, and that having a collaborative meeting between social security supervisors, healthcare providers and patients was experienced to be beneficial. However, all the informants gave examples that only having such meetings would not be enough.

### Relation(ship) with social security supervisor shaping meeting experiences

Different relationship patterns and their influence on the collaborative meetings were talked about by both patients and social security supervisors. Some patients talked about not having any prior relationship with their supervisors before the meetings, where the most important reason was having changed supervisors several times, which they believed affected the continuity of their process.

Some patients who met their supervisor for the first time in the collaborative meeting said they felt uncomfortable discussing their work- or benefit-related plans during the meeting if the supervisor was not familiar with the case. Social security supervisors acknowledged the fragmentation in the services caused by changing supervisors. They, however, suggested that these collaborative meetings could be used as a starting point to build the relationship between them and the patients, as well as reducing the necessity to explain the sickness history several times to different actors working with the same patients.

“It was a nice meeting. It was a completely new job seeker whom I hadn’t met before. We had only communicated digitally. So, she had informed me about the meeting as well. So then… It’s kind of a nice way to… Yeah, she knew I was coming, and then we got to greet each other.” (S1)

The reversed situation was also experienced, where some patients said they felt unsure whether the meetings’ potential could be fully utilized because their current supervisor was soon to be replaced and would then no longer be involved in making decisions about their case. Thus, they said they were afraid that the next supervisor would determine what actions could be taken without having a deeper understanding of the supervisor who had attended the meeting. Supervisors commented that they had a system of having a “transfer meeting” between the old and new supervisors together with the patient and did not see this as a general problem.

“I didn’t quite agree with that conclusion [in the meeting]. … We didn’t really talk any more about it, in that sense. And that was when I thought I should bring it up with my new supervisor.” (P7)

On the other hand, there were examples where patients and supervisors had an ongoing collaboration and a good relationship. This was experienced to open for/result in better discussions about work- or benefit-related opportunities the patients had. Some supervisors highlighted the additional influence such collaborative meetings could have on providing a new perspective on the patients’ lives and a more holistic view on their situation.

“There were things [about the patient] that I wasn’t aware of, and I also didn’t know that it could affect physical pain, you know? … So, you get to know the patient in a slightly different way when you gain that kind of insight [from healthcare providers].” (S4)

### Information exchange or discussion?

From the experiences of participating in the collaborative meetings, it was clear that there were big variations, from the meetings being talked about as being only a place for information sharing to stories of meetings providing the opportunity for good discussions around future plans.

When the meeting was perceived primarily as an information exchange between professionals and the patient, this was often experienced as necessary and appropriate. It was argued that it was important that everyone got the same information, typically information from the consultations the patient had had at the pain clinic. However, some patients and supervisors had expected the meetings to offer more room for discussion on future plans and work or school accommodation. Social security supervisors noted that in some cases, information sharing alone could be sufficient, depending on where the patient was in their process. Some talked about the necessity to use the meetings as a check-in between different actors, as well as with patients to make sure that the already made plan was still applicable considering the patients’ current situation. However, some supervisors felt excluded from the decision-making process, as they were only informed of the conclusion regarding the patient’s care during the meeting, although they might agree with/support the conclusion.

“I didn’t feel that I was able to contribute to or take part in the discussion about the actual plan. For me, it seemed like they only wanted NAV to be involved to ensure that work assessment allowance would continue during the process she was going through. … I wasn’t part of the meeting, really. It was more like, “here’s our plan.” But I wish I had been part of or at least been available for the discussion.” (S5)

Despite talking about the benefit of being informed, some patients said they would have liked to have the opportunity to discuss future plans. Different reasons for this not happening mentioned by both patients and supervisors, were lack of time dedicated to the meetings, having rigid framework for the meetings, as well as supervisors not being engaged enough during the meetings due to changes in supervisors in the near future were mentioned.

“It would have been nice to jump in and say something. I don’t want to entirely blame it on time. Still, it felt like there was a very fixed plan for what we were supposed to go through during the time we had. That made it a bit difficult for me to find the space to add anything or ask questions, because it already felt like they had halfway decided what was going to be said.” (P6)

There were on the other hand patients who said they experienced the meeting an opportunity to discuss the way forward. A typical topic of discussion was about whether the patient should get permanent disability benefit. Both patients and supervisors experienced the meetings to contribute to either clarifying the decision about getting the benefit or ensuring the next steps. An example was a meeting where through discussions about the patient’s current situation and exchange of perspectives over the possibilities, they concluded to put work-oriented measures on pause and get back to them when the patient would have more capacity for them. Some supervisors also said that having open discussions with healthcare providers and being able to ask follow-up questions strengthened their overall understanding of the patients and their situation.

“The role the pain Center has played, especially the psychologist and the psychomotor physiotherapist, has been to help the patient calm down. Like, ‘You can’t do everything you’re physically capable of, because you won’t get better that way. Your body is already working hard.’ So they played an important role in helping us understand what’s too much and what’s too little. And when a specialist says, ‘this isn’t feasible,’ it gives you reassurance as a NAV supervisor. It helps you trust your gut feeling when others confirm what you’ve already sensed.” (S4)

Some supervisors also talked about how such meetings provided an opportunity to explain to both the patients and the healthcare providers about what the regulations and requirements of the process were. A prominent example was related to discussing the needed documentation to assess whether patients were qualified for getting permanent disability benefit and whether these were met.

“I’ve been with NAV for four years and have been receiving what’s called work assessment allowance for four years. We’ve looked into what I can and can’t do [with the pain center], … to see whether I can get disability benefits. Because I’m not capable of working. But since I’m not in what they call active treatment, it becomes very difficult to get approved. So that was actually why NAV was involved too, to find out whether I can get disability benefits or not, and what the process looks like.” (P7)

### Dynamics of collaborative meetings

The informants talk about how having a collaborative meeting between healthcare providers and social security supervisors could have advantages for case management. However, it was experienced that the dynamic of collaborative meetings had been affected by how the meetings were set up.

Both the patients and the supervisors frequently talked about the flow of the meetings. As described in the setting, the common practice was having the social security supervisor participating towards the end of the meeting. This practice was said to disrupt the flow of the meetings in some cases, which resulted in patients becoming unsatisfied with the meeting. Some supervisors also explained how this practice contributed to them not feeling included in the discussions happening during the meeting.

“There was one time I got a question [during the meeting], and I think I spoke for about half a minute, explaining things, and then it popped up on the screen that the supervisor had joined the meeting. But it got interrupted, because there was a bit of distraction and the supervisor had to be brought up to speed on what we had already gone through. And then we never returned to what I was saying.” (P6)

It was also commented on the number of participants. Some patients said they felt overwhelmed due to having several participants in the meeting, which made them reluctant to be active. The reasons given were not feeling comfortable taking up space and asking questions. As described above, some also talked about there not being enough time dedicated to the patients’ opinion. Having more information about what the meetings were planned to focus on and who would be present, were mentioned by supervisors to help better use the potential of the meetings and create a better dialogue. Some supervisors stressed the importance of letting the patients decide who to include in the meetings in general.

“I think maybe it is important with the dialogue the patients have with the specialists themselves, like, ‘Who do you think should be involved in your case?’ You have those who want to include everyone, and then you have those who might not see it as necessary and want to have a bit more control themselves. They want to have a say about who joins. After all, it’s their case, and they’re supposed to be the ones in charge of it.” (S4)

Some patients and supervisors also talked about how having digital meetings can influence dynamics. For some supervisors, it was experienced to be difficult to join the meeting digitally after the others and not being properly introduced to the people in the meeting. However, the supervisors highlighted the importance of considering patients’ preference for deciding to have the meetings physically or digitally.

“I can sort of understand it, because there are a lot of different healthcare providers involved, and they do have a different role than I do. But it still felt a bit unnatural that I was on Teams while they were all sitting there together as a group.” (S5)

### Collaboration extending beyond the meeting

Both supervisors and patients reported a need for more discussions and clarification of issues in additional follow-up meetings. Several topics for such meetings were mentioned including improvement of the relationship between patients and their social security supervisor, discussing the opportunities for the patient based on what happened during the collaborative meeting, or reassuring that the patient agreed with the conclusions that were made during the collaborative meeting.

Regarding the need for a clearer plan after the meeting, one patient used the word being “lost” after the meeting concerning the direction their case would go in with social security service. Social security supervisors agreed with the importance of using the meetings to decide and/ or clarify the follow-up plans. In addition, several supervisors talked about the need to decide who had the responsibility for taking initiatives for the planned follow-up activities, where they believed both patients and supervisors share the responsibility in the matter.

“But at the same time, the patient also has a responsibility to follow up on their own case. So, it’s also a matter of whether they are good enough at asking. We can slip up too, we can make mistakes as NAV supervisors, right? But then it’s important that the patient reaches out themselves and says, ‘Hey, what happened with my case?’ And we often find that this doesn’t happen.” (S4)

Some supervisors mentioned the difference in the amount of contact needed in different stages of the cases. One example was when the conclusion of the meeting was to apply for permanent disability benefit. In such cases, close follow-up was expressed as not necessary, and any contact after the meeting would serve more as check-ins to ensure that the process was moving forward as planned.

“We’ve told the patient that maybe in five or six years her situation might be completely different, once she’s gotten things more in order. And this is a young person who’s in the early stages of building a life with a partner and a home and children. But it’s hard to imagine managing a job, health issues, and children all at once, you know? So, you must look at the whole picture. But we have a very good and positive relationship, she and I. So, she calls if something comes up, and we sort it out.” (S4)

On the other hand, changing supervisors in the near future or having an ongoing discussion about different aspects of the case were mentioned to be the reasons for close follow-up and contact between the patient and the supervisor.

“My role as a supervisor, at least, is about providing close follow-up when there’s a need for it. And then at other times, when someone is going through processes that are so demanding that they don’t need [extra load], that’s not when NAV should be too involved. But it’s important to maintain contact so we can assess when it’s the right time to step in again.” (S1)

## Discussion

The patients and social security supervisors experienced collaboration between social security service and healthcare service through meetings to be useful. However, many emphasized that the quality of these meetings was influenced by the (lack of) preexisting relationship between patients and their social security supervisors. In some cases, the meetings were limited to the exchange of information, while in others, they created a space for discussion and the development of a shared understanding of the situation. Some informants noted that the structure of the meetings could make it difficult to maintain a dialogue. Some patients also mentioned that the presence of many participants in the meetings could feel overwhelming. Although most informants valued the opportunity to take part in these collaborative meetings, they also acknowledged that effective collaboration begins ahead of and extends beyond a single meeting and should be understood as part of a broader, ongoing process.

### Relationship building

The collaborative meetings in this study were conducted digitally. While digital meetings and video consultations in healthcare can improve accessibility, particularly for patients who are unable to attend in person due to geographic distance or health conditions [37], this format may also negatively impact relationship-building between patients and service providers, as well as among members of the provider team [38, 39]. Previous research has emphasized the importance of preexisting relationships between patients and service providers in the context of digital interactions [40]. This is in line with the current findings that the quality of the prior relationship between patients and social security supervisors was experienced by both as contributing to the quality of collaboration.

There was a difference in the patients and supervisors’ view of the situation when a patient meets their supervisor for the first time at the collaborative meeting, where the patient talked about this as a negative experience, while some supervisors said it could be an opportunity to initiate relationships. While the latter may reflect the possibility for supervisors to get information about the case, thinking of the meeting as a place for initiating a relationship seems unrealistic as there is very limited time and sensitive topics to be discussed. As described in the literature, collaboration tends to be more effective when relationship-building is treated as an ongoing process rather than limited to specific events [41]. A continuous approach increases the likelihood of building trust, which is often described as an important factor in facilitating cross-sectoral collaboration between social security and healthcare service [25, 26].

A key reason for the lack of such relationships between patients and social security supervisors, as reported by informants in this study, was the frequent changes in supervisors experienced by patients, due to the organizational structure of the social security service in Norway. This often led to fragmented communication and a sense of constantly starting over. Thus, digital meetings may be more effective when a relationship between the patient and their social security supervisor has already been established. In addition, it may be particularly important to invest additional effort in preparing participants for collaborative meetings, especially when they are unfamiliar with their supervisors. Previous research on interprofessional teams has shown that adequate preparation, clear information and clarification of roles before meetings are important for supporting user participation and collaboration between service providers [42, 43]. This might suggest that preparation could serve not only as a practical function but also as a relational one, helping participants to feel informed and aligned in their understanding of the meeting’s purpose. Clear preparation could reduce uncertainty, foster a sense of involvement, and ultimately contribute to more constructive and goal-oriented discussions.

### From information exchange to meaningful dialogue

A key insight from the findings in this study is that the experience of the meetings varies from mere information-sharing to those meetings that foster genuine dialogue and joint decision-making. While information exchange is necessary, it is insufficient for addressing the complex and changing needs of patients with chronic conditions [44, 45]. Social security supervisors who had long-standing relationships with their clients or patients described being better positioned to advocate for them and interpret their needs during meetings, which suggests that continuity in relationships can be important for creating space in collaborative meetings to move beyond mere information exchange and toward discussions about shared reflection and forward planning. This finding aligns with recent literature emphasizing that good routines for communication and a history of collaboration between healthcare and non-healthcare organizations can influence the dynamics of cross-sectoral collaboration [27].

In some cases, even when meetings included conversations around planning forward, patients perceived a lack of involvement, as decisions appeared to have been made beforehand. This resonates with findings showing that, although professionals often believe they are practicing shared decision-making, patients frequently feel excluded from the process [46, 47]. Such unsatisfactory experiences may also reflect a broader issue about the limited involvement of patients in the design and development of collaborative services [17]. Supervisors also expressed frustration at being excluded from the planning processes. This limited involvement undermines the potential for collaboration to generate integrated, person-centered solutions, as it has been shown that cross-sectoral interventions involving various actors contribute to improving functional status [22]. Thus, to support a move from procedural to participatory meetings, structural changes seem to be necessary. As mentioned earlier in the discussion, potential solutions include preparing participants in advance of meetings, allocating sufficient number of meetings and time during meetings and using facilitation techniques that promote equitable dialogue and shared decision-making.

### Sustaining collaboration beyond the meeting room

While collaborative meetings were experienced as important touchpoints, their long-term value depends on what happens prior to and after the meetings. The findings in this study showed that both patients and social security supervisors often experienced a lack of clarity regarding follow-up responsibilities, which could lead to confusion, delays, and in some cases, disengagement. Recent literature confirms that without clear governance and accountability structures; collaborative efforts often fail to translate into lasting outcomes, and while many local collaborations between healthcare and social services are initiated with enthusiasm, they frequently lack the operational infrastructure to sustain collaboration over time [48]. Similarly, it is emphasized that follow-up processes must be embedded in routine workflows and not left to individual discretion [49].

Supervisors in this study mentioned that the follow-up intensity from social security service would vary depending on patients’ situations and differ for each patient. For example, if the conclusion of the meeting was that the patient would apply for permanent disability benefit, less frequent contact was needed. However, in more dynamic or uncertain cases, closer follow-up was essential. This aligns with findings indicating that collaboration must be flexible and responsive to the changing needs of patients, as rigid or one-size-fits-all schedules fail to address fluctuating care demands [50]. Patients, on the other hand, often felt unsure about the nature and extent of follow-up they should expect. Although supervisors indicated that the responsibility for initiating follow-up also could be put on patients, this ambiguity and the burden of responsibility might be particularly disempowering for individuals with chronic fatigue, who already spend significant time and energy struggling with the uncertainties regarding treatment options and lack of clear guidance [51, 52].

### Strengths and limitations

A key strength of this study lies in its inclusion of both patients and social security supervisors, offering a dual perspective on cross-sectoral collaboration in the management of chronic fatigue. This approach enabled a richer understanding of the collaborative process, particularly by allowing supervisors to reflect on and respond to patient experiences. Such triangulation of perspectives adds depth to the findings and enhances the credibility of the analysis. Additionally, this is, to our knowledge, the first study to explore collaborative meetings between healthcare and social security services specifically for patients with chronic fatigue, contributing novel insights to an under-researched area.

However, the methodological choices also shaped the findings in specific ways. While supervisors were invited to comment on patient experiences, the reverse was not part of the study design. Including patient reflections on supervisors’ perspectives could have revealed additional dynamics or tensions in the collaborative process. In addition, patients and social security supervisors were not always recruited from the same meeting. Including perspectives from the same meeting could have provided additional insights into the topic. Furthermore, the study was conducted in a single geographical region in Norway, which may limit the transferability of the findings to other contexts with different organizational structures or collaboration practices. The informants were mostly women, which reflects the gender distribution in the patient population. However, this may have influenced the perspectives represented in the data. Finally, the perspectives of other key participants in the meetings, such as general practitioners or specialist healthcare providers, were not included, which may have left out important viewpoints on the collaborative process.

## Conclusion

This study highlights the value and complexity of cross-sectoral collaboration between healthcare and social security services in the case management of patients with chronic fatigue. Collaborative meetings were generally experienced as beneficial by both patients and social security supervisors, particularly when they facilitated mutual understanding and joint planning. However, the effectiveness of these meetings was perceived to be influenced by several factors, including the quality of the relationship between meeting participants, the structure and timing of the meetings, and the extent to which participants felt included in the dialogue.

The findings underscore that meaningful collaboration cannot be reduced to a single meeting but must be understood as part of a broader, ongoing process. Structural and procedural adjustments, including ensuring continuity in supervisory relationships, preparing participants in advance, and clarifying follow-up responsibilities after the meeting, are suggested as needed actions to support more integrated and person-centered care.

## Supplementary Information

Below is the link to the electronic supplementary material.

Supplementary Material 1

Supplementary Material 2

## Data Availability

The data supporting the conclusions of this article is not openly available, but the anonymized transcripts are available from the corresponding author upon reasonable request. The data can be found at the Department of Public Health and Nursing at the Norwegian University of Science and Technology in Trondheim, Norway.

## Abbreviations

NAV: Norwegian Labour and Welfare Administration
ME/CFS: Myalgic Encephalomyelitis/Chronic Fatigue Syndrome

## References

[1] Glette M, Stiles TC, Woodhouse A, Nilsen TIL, Landmark T. Chronic fatigue in the general population: Prevalence, natural course and associations with chronic pain (the HUNT pain study). Eur J Pain. 2024;28(10):1762–71. 38940382 10.1002/ejp.2307

[2] Institute of Medicine. Beyond myalgic encephalomyelitis/chronic fatigue syndrome: redefining an illness. Washington, D.C.: National Academies; 2015. 25695122

[3] Brurberg KG, Fønhus MS, Larun L, Flottorp S, Malterud K. Case definitions for chronic fatigue syndrome/myalgic encephalomyelitis (CFS/ME): a systematic review. BMJ Open. 2014;4(2):e003973. 24508851 10.1136/bmjopen-2013-003973 PMC3918975

[4] White RA, Zhang C, Valcarcel Salamanca B, Angelsen A, Zakiudin DP, Andries A, et al. Aberrations in medically certified sick leave and primary healthcare consultations in Norway in 2023 compared to pre-COVID-19-pandemic trends. Arch Public Health. 2024;82(1):187. 39438966 10.1186/s13690-024-01411-4 PMC11495095

[5] Stevelink SAM, Mark KM, Fear NT, Hotopf M, Chalder T. Chronic fatigue syndrome and occupational status: a retrospective longitudinal study. Occup Med (Lond). 2022;72(3):177–83. 34865116 10.1093/occmed/kqab170

[6] Bartlett C, Hughes JL, Miller L. Living with myalgic encephalomyelitis/chronic fatigue syndrome: experiences of occupational disruption for adults in Australia. Br J Occup Ther. 2022;85(4):241–50. 40337214 10.1177/03080226211020656 PMC12033770

[7] Castro-Marrero J, Faro M, Zaragozá MC, Aliste L, de Sevilla TF, Alegre J. Unemployment and work disability in individuals with chronic fatigue syndrome/myalgic encephalomyelitis: a community-based cross-sectional study from Spain. BMC Public Health. 2019;19(1):840. 31253111 10.1186/s12889-019-7225-z PMC6599355

[8] Palstam A, Mannerkorpi K. Work ability in fibromyalgia: an update in the 21st century. Curr Rheumatol Rev. 2017;13(3):180–7. 28464770 10.2174/1573397113666170502152955 PMC5759171

[9] Lim EJ, Ahn YC, Jang ES, Lee SW, Lee SH, Son CG. Systematic review and meta-analysis of the prevalence of chronic fatigue syndrome/myalgic encephalomyelitis (CFS/ME). J Transl Med. 2020;18(1):100. 32093722 10.1186/s12967-020-02269-0 PMC7038594

[10] Bakken IJ, Tveito K, Gunnes N, Ghaderi S, Stoltenberg C, Trogstad L, et al. Two age peaks in the incidence of chronic fatigue syndrome/myalgic encephalomyelitis: a population-based registry study from Norway 2008–2012. BMC Med. 2014;12:167. 25274261 10.1186/s12916-014-0167-5 PMC4189623

[11] Blease C, Carel H, Geraghty K. Epistemic injustice in healthcare encounters: evidence from chronic fatigue syndrome. J Med Ethics. 2017;43(8):549–57. 27920164 10.1136/medethics-2016-103691

[12] Colombo B, Zanella E, Galazzi A, Arcadi P. The experience of stigma in people affected by fibromyalgia: A metasynthesis. J Adv Nurs. 2025;81(10):6317–32. 39835578 10.1111/jan.16773 PMC12460976

[13] Kachaner A, Harim M, Combier A, Trouvin AP, Avouac J, Ranque B, et al. Management perspectives from patients with fibromyalgia experiences with the healthcare pathway: a qualitative study. Front Med (Lausanne). 2023;10:1231951. 38105901 10.3389/fmed.2023.1231951 PMC10722233

[14] Brimmer DJ, Fridinger F, Lin J-MS, Reeves WC. US healthcare providers’ knowledge, attitudes, beliefs, and perceptions concerning chronic fatigue syndrome. BMC Fam Pract. 2010;11:1–15. 20406491 10.1186/1471-2296-11-28 PMC2875206

[15] Mengshoel AM, Sim J, Ahlsen B, Madden S. Diagnostic experience of patients with fibromyalgia - A meta-ethnography. Chronic Illn. 2018;14(3):194–211. 28762775 10.1177/1742395317718035

[16] Hansen AH, Lian OS. How do women with chronic fatigue syndrome/myalgic encephalomyelitis rate quality and coordination of healthcare services? A cross-sectional study. BMJ Open. 2016;6(4):e010277. 27044578 10.1136/bmjopen-2015-010277 PMC4823449

[17] Melby L, Nair RD. We have no services for you… so you have to make the best out of it’: A qualitative study of myalgic Encephalomyelitis/Chronic fatigue syndrome patients’ dissatisfaction with healthcare services. Health Expect. 2024;27(1):e13900. 37905602 10.1111/hex.13900 PMC10726260

[18] Åsbring P, Närvänen A-L. Women’s experiences of stigma in relation to chronic fatigue syndrome and fibromyalgia. Qual Health Res. 2002;12(2):148–60. 11837367 10.1177/104973230201200202

[19] Thomas R, Hughes J, Kotzur C. What is the occupational impact of myalgic encephalomyelitis/chronic fatigue syndrome for adults living in australia? Br J Occup Ther. 2024;87(10):636–44. 40343209 10.1177/03080226241254720 PMC11887866

[20] Vink M, Vink-Niese F. Work rehabilitation and medical retirement for myalgic encephalomyelitis/chronic fatigue syndrome patients. a review and appraisal of diagnostic strategies. Diagnostics (Basel). 2019;9(4). 10.3390/diagnostics9040124 PMC6963831 31547009

[21] Geraghty KJ, Blease C. Myalgic encephalomyelitis/chronic fatigue syndrome and the biopsychosocial model: a review of patient harm and distress in the medical encounter. Disabil Rehabil. 2019;41(25):3092–102. 29929450 10.1080/09638288.2018.1481149

[22] Nazarov S, Manuwald U, Leonardi M, Silvaggi F, Foucaud J, Lamore K, et al. Chronic diseases and employment: which interventions support the maintenance of work and return to work among workers with chronic illnesses? A systematic review. Int J Environ Res Public Health. 2019;16(10). 10.3390/ijerph16101864 PMC6572561 31137817

[23] Hansen AH, Lian OS. Experiences of general practitioner continuity among women with chronic fatigue syndrome/myalgic encephalomyelitis: a cross-sectional study. BMC Health Serv Res. 2016;16(1):650. 27842587 10.1186/s12913-016-1909-1 PMC5109710

[24] Nolte E, Knai C. Assessing chronic disease management in European health systems. Country reports: World Health Organization; 2015. 29035490

[25] Poulsen RM, Pii KH, Eplov LF, Meijer M, Bultmann U, Christensen U. Developing interpersonal trust between service users and professionals in integrated services: compensating for latent Distrust, vulnerabilities and uncertainty shaped by organisational context. Int J Integr Care. 2021;21(3):1. 34248445 10.5334/ijic.5599 PMC8252972

[26] Schultz R, Brostrøm Kousgaard M, Davidsen AS. We have two different agendas: the views of general practitioners, social workers and hospital staff on interprofessional coordination for patients with chronic widespread pain. J Interprof Care. 2021;35(2):284–92. 32297802 10.1080/13561820.2020.1749576

[27] Alderwick H, Hutchings A, Briggs A, Mays N. The impacts of collaboration between local health care and non-health care organizations and factors shaping how they work: a systematic review of reviews. BMC Public Health. 2021;21(1):753. 33874927 10.1186/s12889-021-10630-1 PMC8054696

[28] Brijnath B, Mazza D, Singh N, Kosny A, Ruseckaite R, Collie A. Mental health claims management and return to work: qualitative insights from Melbourne, Australia. J Occup Rehabil. 2014;24:766–76. 24647855 10.1007/s10926-014-9506-9

[29] MacEachen E, Clarke J, Franche R-L, Irvin E, Group W-bRtWLR. Systematic review of the qualitative literature on return to work after injury. Scand J Work Environ Health. 2006;32(4):257–69. 16932823

[30] Tong A, Sainsbury P, Craig J. Consolidated criteria for reporting qualitative research (COREQ): a 32-item checklist for interviews and focus groups. Int J Qual Health Care. 2007;19(6):349–57. 17872937 10.1093/intqhc/mzm042

[31] Langvarig utmattelse uten kjent årsak inkludert CFS/ME -. Nasjonalt pasientforløp [Chronic fatigue without known cause including CFS/ME - National patient pathway]: Helsedirektoratet 2022 [cited 2025 July 20]. Available from: https://www.helsedirektoratet.no/horinger/langvarig-utmattelse-uten-kjent-arsak-inkludert-cfs-me-nasjonalt-pasientforlop

[32] The Norwegian Social Insurance Scheme: Norwegian Ministry of Labour and Social Inclusion. 2025 [cited 2025 July 20]. Available from: https://www.regjeringen.no/contentassets/7404ed915a2b492fa10288752e82b132/the-norwegian-social-insurance-scheme-2025.pdf

[33] Nylenna M. Helsetjenesten i Norge: et overblikk [The health service in Norway: an overview]: Gyldendal akademisk; 2014.

[34] Norwegian Labour and Welfare Adminstration. Informasjonsutveksling mellom NAV og fastleger [Information exchange between NAV and general practitioners]. 2021.

[35] Saunders B, Sim J, Kingstone T, Baker S, Waterfield J, Bartlam B, et al. Saturation in qualitative research: exploring its conceptualization and operationalization. Qual Quant. 2018;52(4):1893–907. 29937585 10.1007/s11135-017-0574-8 PMC5993836

[36] Malterud K. Systematic text condensation: a strategy for qualitative analysis. Scand J Public Health. 2012;40(8):795–805. 23221918 10.1177/1403494812465030

[37] Bavngaard MV, Lüchau EC, Hvidt EA, Grønning A. Exploring patient participation during video consultations: A qualitative study. Digit Health. 2023;9:20552076231180682. 37325071 10.1177/20552076231180682 PMC10265318

[38] Song H, Ryan M, Tendulkar S, Fisher J, Martin J, Peters AS, et al. Team dynamics, clinical work satisfaction, and patient care coordination between primary care providers: A mixed methods study. Health Care Manage Rev. 2017;42(1):28–41. 26545206 10.1097/HMR.0000000000000091

[39] Andreadis K, Muellers K, Ancker JS, Horowitz C, Kaushal R, Lin JJ. Telemedicine impact on the Patient-Provider relationship in primary care during the COVID-19 pandemic. Med Care. 2023;61(Suppl 1):S83–8. 36893423 10.1097/MLR.0000000000001808 PMC9994565

[40] Wanderås MR, Abildsnes E, Thygesen E, Martinez SG. Video consultation in general practice: a scoping review on use, experiences, and clinical decisions. BMC Health Serv Res. 2023;23(1):316. 36997997 10.1186/s12913-023-09309-7 PMC10063329

[41] D’Amour D, Ferrada-Videla M, San Martin Rodriguez L, Beaulieu MD. The conceptual basis for interprofessional collaboration: core concepts and theoretical frameworks. J Interprof Care. 2005;19(Suppl 1):116–31. 16096150 10.1080/13561820500082529

[42] Donnelly S, Butler P, O’Loughlin A, Russell E, Carroll P. Interdisciplinary team members’ experiences of family meetings in an Irish rehabilitation hospital: a social work action-research project. Eur Social Work Res. 2024;2(1):3–23.

[43] van Dongen JJJ, Habets IGJ, Beurskens A, van Bokhoven MA. Successful participation of patients in interprofessional team meetings: a qualitative study. Health Expect. 2017;20(4):724–33. 27714904 10.1111/hex.12511 PMC5513000

[44] Moen EÅ, Larsen IB, Walseth LT. Employment and mental health recovery: revealing unused potential in multi-agency meetings. NJWEL. 2023;2(1):5–19.

[45] Haghshenas M, Steinsbekk A, Hara KW. Experiences of cross-sectoral collaboration between social security service and healthcare service for patients with chronic pain - a qualitative study. Scand J Pain. 2025;25(1). 10.1515/sjpain-2024-0057 40226932

[46] Waddell A, Lennox A, Spassova G, Bragge P. Barriers and facilitators to shared decision-making in hospitals from policy to practice: a systematic review. Implement Sci. 2021;16(1):74. 34332601 10.1186/s13012-021-01142-y PMC8325317

[47] Moleman M, Regeer BJ, Schuitmaker-Warnaar TJ. Shared decision-making and the nuances of clinical work: Concepts, barriers and opportunities for a dynamic model. J Eval Clin Pract. 2021;27(4):926–34. 33164316 10.1111/jep.13507 PMC8359199

[48] Alderwick H, Hutchings A, Mays N. Cross-sector collaboration to reduce health inequalities: a qualitative study of local collaboration between health care, social services, and other sectors under health system reforms in England. BMC Public Health. 2024;24(1):2613. 39334058 10.1186/s12889-024-20089-5 PMC11438096

[49] Mejdahl CT, Schougaard LMV, Hjollund NH, Riiskjær E, Lomborg K. Exploring organisational mechanisms in PRO-based follow-up in routine outpatient care - an interpretive description of the clinician perspective. BMC Health Serv Res. 2018;18(1):546. 30001729 10.1186/s12913-018-3352-y PMC6044066

[50] Persson MH, Søndergaard J, Mogensen CB, Skjøt-Arkil H, Andersen PT. Healthcare professionals’ experiences and attitudes to care coordination across health sectors: an interview study. BMC Geriatr. 2022;22(1):509. 35729544 10.1186/s12877-022-03200-6 PMC9210644

[51] Hasan Z, Kuyvenhoven C, Chowdhury M, Amoudi L, Zeraatkar D, Busse JW, et al. Patient perspectives of recovery from myalgic encephalomyelitis/chronic fatigue syndrome: an interpretive description study. J Eval Clin Pract. 2024;30(2):234–42. 37927138 10.1111/jep.13938

[52] McDermott C, Lynch J, Leydon GM. Patients’ hopes and expectations of a specialist chronic fatigue syndrome/ME service: a qualitative study. Fam Pract. 2011;28(5):572–8. 21555341 10.1093/fampra/cmr016

