# Supplementary material for: Information exchange or discussion? A qualitative study on cross-sectoral collaboration between social security service and healthcare service for patients with chronic fatigue
Source: BMC Health Serv Res. 2025 Dec 9;26:63. doi: 10.1186/s12913-025-13857-5 (PMC12801834; doi:10.1186/s12913-025-13857-5)
Supplement: Supplementary file 1 — Supplementary Material 1 [file 12913_2025_13857_MOESM1_ESM.docx]

**Interview guide patients**

1. What are your experiences with the cross-sectoral collaborative meeting you participated in at the pain center, starting from when you first learned it would take place?
2. How did you experience the collaboration between your social security supervisor and healthcare providers?
