# Supplementary material for: Information exchange or discussion? A qualitative study on cross-sectoral collaboration between social security service and healthcare service for patients with chronic fatigue
Source: BMC Health Serv Res. 2025 Dec 9;26:63. doi: 10.1186/s12913-025-13857-5 (PMC12801834; doi:10.1186/s12913-025-13857-5)
Supplement: Supplementary file 2 — Supplementary Material 2 [file 12913_2025_13857_MOESM2_ESM.docx]

**Interview guide for social security supervisors**

1. What are your experiences with having cross-sectoral collaboration with specialist healthcare in follow-up of users?
2. How did you experience participating in the cross-sectoral collaborative meeting at the pain center with patients, general practitioners and specialist healthcare providers?
3. What are your opinions about the following statements, which are patients’ experiences from participating in the mentioned cross-sectoral collaborative meetings at the pain center?
   1. Having to change social security supervisors several times
   2. Experiencing the meetings as primarily for information exchange rather than treatment planning
   3. Lacking clear follow-up from social security service after the meetings
   4. Feeling uncomfortable sharing about personal feelings in this setting
   5. Feeling outnumbered or overpowered due to being alone while facing several professionals
